# Regulated Forms of Cell Death in Fungi

**DOI:** 10.3389/fmicb.2017.01837

**Published:** 2017-09-21

**Authors:** A. Pedro Gonçalves, Jens Heller, Asen Daskalov, Arnaldo Videira, N. Louise Glass

**Affiliations:** ^1^Plant and Microbial Biology Department, University of California, Berkeley Berkeley, CA, United States; ^2^Instituto de Ciências Biomédicas de Abel Salazar, Universidade do Porto Porto, Portugal; ^3^I3S - Instituto de Investigação e Inovação em Saúde Porto, Portugal

**Keywords:** programmed cell death, filamentous fungi, calcium, ROS, metacaspases, NLR, BCL-2 family

## Abstract

Cell death occurs in all domains of life. While some cells die in an uncontrolled way due to exposure to external cues, other cells die in a regulated manner as part of a genetically encoded developmental program. Like other eukaryotic species, fungi undergo programmed cell death (PCD) in response to various triggers. For example, exposure to external stress conditions can activate PCD pathways in fungi. Calcium redistribution between the extracellular space, the cytoplasm and intracellular storage organelles appears to be pivotal for this kind of cell death. PCD is also part of the fungal life cycle, in which it occurs during sexual and asexual reproduction, aging, and as part of development associated with infection in phytopathogenic fungi. Additionally, a fungal non-self-recognition mechanism termed heterokaryon incompatibility (HI) also involves PCD. Some of the molecular players mediating PCD during HI show remarkable similarities to major constituents involved in innate immunity in metazoans and plants. In this review we discuss recent research on fungal PCD mechanisms in comparison to more characterized mechanisms in metazoans. We highlight the role of PCD in fungi in response to exogenic compounds, fungal development and non-self-recognition processes and discuss identified intracellular signaling pathways and molecules that regulate fungal PCD.

## Introduction

**Figure 1:**
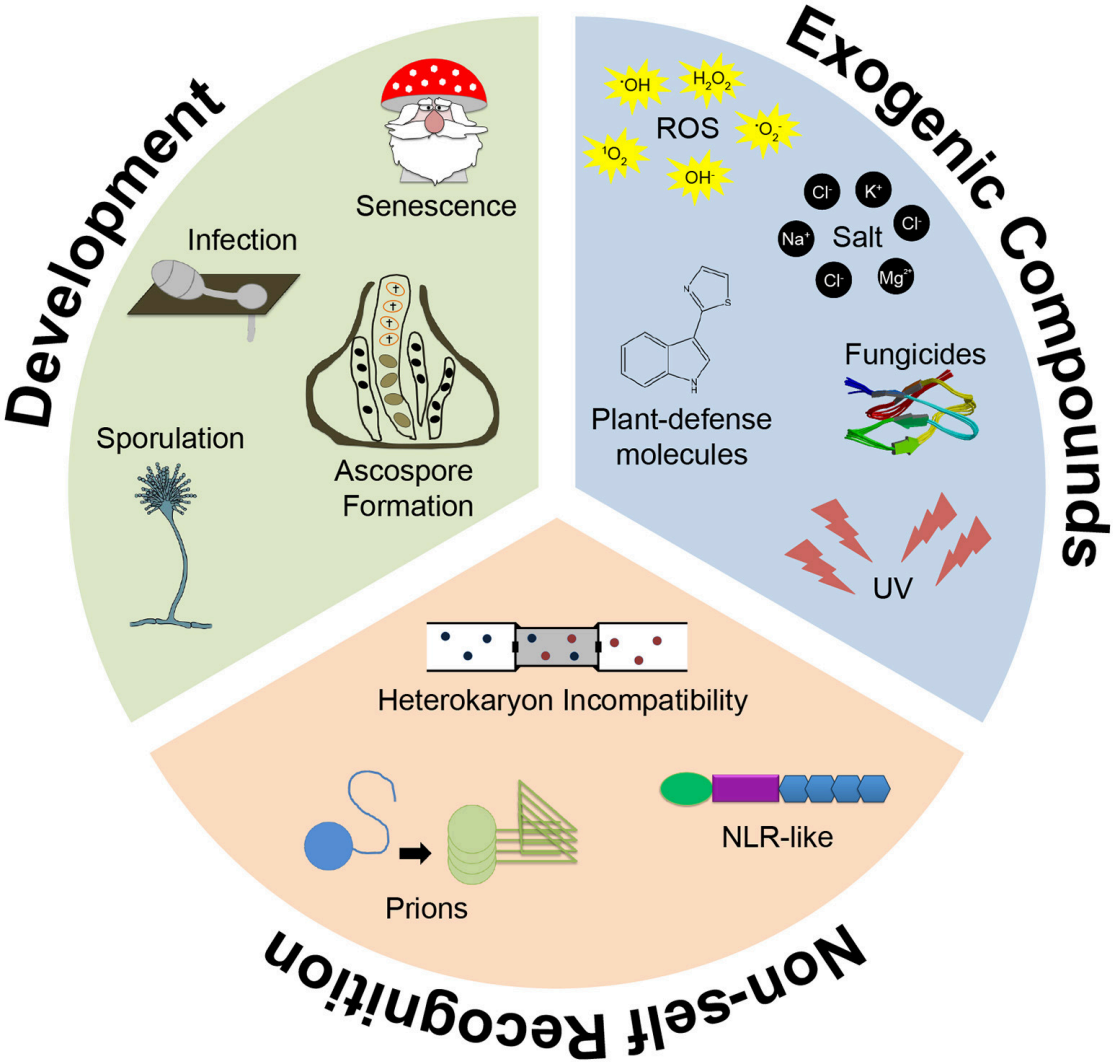
Programmed Cell Death (PCD) in filamentous fungi. PCD has been reported to occur in filamentous fungi in response to various exogenic compounds, such as plant defense molecules (e.g., camalexin), toxins and fungicides (e.g., Penicillium AntiFungal protein), during non-self-recognition and heterokaryon incompatibility and during developmental programs throughout the fungal life cycle, including morphogenesis associated plant infection.

We have all heard the phrase “death is a part of life,” which most often is referred to in connection with the human lifespan. However, this statement can be applied to all organisms when we think of death in a broader sense, i.e., cell death. Many cells die in a regulated manner controlled by an intracellular process termed programmed cell death (PCD). In general, some form of PCD occurs in all domains of life, from bacteria to higher eukaryotes (Engelberg-Kulka et al., 2006; Fuchs and Steller, 2011; van Doorn, 2011). In animals, the term PCD is mostly associated with developmental processes such as modeling of organs, regulation of cell number or deletion of certain macrostructures and removal of defective and potentially harmful cells (Fuchs and Steller, 2011). At the cellular level PCD is often mediated by an apoptotic mechanism associated with the externalization of phosphatidylserine to the outer leaflet of the plasma membrane, DNA fragmentation, cytochrome C release from mitochondria and caspase activation (Fuchs and Steller, 2015). In this review we highlight the role of PCD in fungi and summarize the knowledge on molecular mediators of regulated death in these organisms, with a special emphasis on filamentous species (Figure 1).

### General considerations on cell death

There are many ways for a cell to die. Death can occur in response to physicochemical and mechanical triggers or it can occur in a controlled way by a genetically encoded apparatus. Classically, there have been three main categories of cell death: autophagy, apoptosis, and necrosis. Apoptosis and autophagy are genetically regulated PCD reactions that allow the orderly degradation and recycling of cellular components whereas necrosis has been classically defined as a more abrupt type of death driven by physical or chemical injuries. Advances in research on cell death have revealed an immense complexity of these processes, which has blurred the lines between what was traditionally defined as uncontrolled vs. controlled death. Additional forms of programmed cell death have been defined including the finding that cells can execute necrosis in a programmed fashion (necroptosis) or die in a regulated manner in response to an infection caused by intracellular pathogens (pyroptosis). To help researchers to discriminate and define different aspects of cell death the Nomenclature Committee on Cell Death (NCCD) has released recommendations for the classification of cell death to bring consensus in the field (Kroemer et al., 2005, 2009; Galluzzi et al., 2012, 2015).

The concept of cell death as a programmed event was initially suggested in the 19th century, when Carl Vogt described cell death during amphibian metamorphosis (Vogt, 1842; Clarke and Clarke, 1996). Ever since, PCD in multicellular metazoans was generally referred to as a developmental strategy to remove unwanted cells and make way for cellular remodeling and differentiation without causing an inflammatory response. The first description of apoptosis dates back to the early 1970s when it was defined as a “mechanism of controlled cell deletion, which appears to play a complementary but opposite role to mitosis in the regulation of animal cell populations” (Kerr et al., 1972). Subsequent genetic studies in the nematode *Caenorhabditis elegans* identified genes dedicated to the developmental death program and its control (Ellis and Horvitz, 1986). Some of these genes are homologous to mammalian genes (Yuan et al., 1993; Hengartner and Horvitz, 1994) and their roles in cell death are largely conserved in metazoans. The importance of PCD correlates with the complexity of the organism. For example, PCD-deficient nematodes have a normal lifespan in the laboratory, even though they have about 15% more cells and function less well than wild-type worms (Ellis et al., 1991). However, PCD-deficient flies die early in development (White et al., 1994), and mice with inhibited apoptosis die perinatally with a vast excess of cells in their central nervous system (Kuida et al., 1996). Over the past two decades, substantial progress has been made in the understanding of the intracellular molecular mechanisms of PCD and its control in metazoans (reviewed in Oberst et al., 2008). Key enzymes involved are specific proteases (caspases) that cleave target proteins at specific aspartic acid residues. Caspases exist as inactive precursors within cells and their activation occurs by conserved mechanisms (oligomerization, cleavage of regulatory pro-domains, and/or dissociation from endogenous inhibitors) and are subject to strict cellular regulation. Limited proteolysis of certain substrates by caspases activate specific downstream signaling pathways ensuring that the cellular components are degraded in a controlled manner to avoid damage of surrounding tissue (Lamkanfi et al., 2007; Pop and Salvesen, 2009; Julien and Wells, 2017; Man et al., 2017). Soon after caspases were identified as major regulators of cell death in metazoans, a hunt for homologous peptidases in other kingdoms was initiated, with distantly related caspase-like proteins (metacaspases) identified in plants, fungi, and protozoa (Uren et al., 2000). However, although metacaspases seem to play some role during cell death in non-metazoans, it is still unclear if they function in comparable fashion as caspases to regulate the transition to cell death (Tsiatsiani et al., 2011).

## The multiple manifestations of PCD in fungi

In the unicellular yeast, *Saccharomyces cerevisiae*, PCD was reported in the 1990s in a temperature-sensitive mutant of *cdc48* (*cdc48*^*S565G*^), which showed an endoplasmic reticulum-localized ATPase, externalization of phosphatidylserine to the outer leaflet of the plasma membrane, and DNA fragmentation and condensation under non-permissive conditions (Madeo et al., 1997). PCD has also been reported in the fission yeast *Schizosaccharomyces pombe*, where it has been associated with perturbation of lipid metabolism, defective DNA replication/mitotic entry, and chronological/replicative aging (Low and Yang, 2008). Despite many additional observations of PCD-like phenotypes in unicellular fungi (Falcone and Mazzoni, 2016), the degree of similarity between fungal and metazoan cell death is still controversial. Coined as *phenoptosis* by Skulachev (1999), cell death of unicellular organisms means organismal death and therefore lacks a developmental aspect. However, the terms *apoptotic-like cell death* or *programmed cell death* have still been employed when dying fungal cells exhibit morphological and biochemical features of PCD including DNA fragmentation and condensation, cell shrinkage, phosphatidylserine exposure, reactive oxygen species (ROS) production, mitochondrial membrane depolarization, reduced glutathione (GSH) efflux and caspase-like activity (Almeida et al., 2008; Low and Yang, 2008; Sharon et al., 2009; Carmona-Gutierrez et al., 2010a; Ramsdale, 2012). Furthermore, cells of yeast colonies form social communities (Palkova and Vachova, 2006) that undergo a differentiation-like program involving regulated cell death (Vopalenska et al., 2005). For example, if a dead zone at the colony center is removed, growth reduction occurs in cells at the periphery of the colony, suggesting that cell death occurring at the center of the colony supports growth of the whole colony (Longo et al., 2005; Vopalenska et al., 2005; Sukhanova et al., 2012). It has been hypothesized that cell density is the threshold factor that determines whether DNA mutational load and the nutritional environment are at a critical value, leading to cell death of parts of the population (Longo et al., 2005; Severin et al., 2008). In line with these findings, both chronological and replicative aging result in the appearance of apoptotic-like markers in *S. cerevisiae* and *S. pombe* (Laun et al., 2001; Herker et al., 2004; Low and Yang, 2008). For example, in chronological aging, a small subpopulation of *S. cerevisiae* cells are able to regrow after more than 90% of the population has died (Fabrizio et al., 2004), possibly due to the release of substances from dying cells that alter the composition of the growth medium (Longo et al., 2005). A recent report also showed the involvement of programmed cell death in yeast gametogenesis. In a process called *programmed nuclear destruction* cells develop aborted meiotic products to control the spore number, which apparently benefits sibling cells (Eastwood et al., 2012). PCD has also been implicated as a key evolutionary event during the transition from uni- to multicellularity in yeast cells. For example, when the ability to form primitive multicellular structures was selected for in yeast cells (through a sedimentation strategy), the development of cell clusters leading to a snowflake-type phenotype was associated with high rates of cell death. Apoptotic cells in the clusters may generate “weak links” that allow small branches to separate from large clusters which can increase a cluster's fecundity (Ratcliff et al., 2012).

### PCD during reproduction

Due to their multicellular nature and higher complexity, PCD plays a more diverse role in filamentous fungi as compared to unicellular yeast (Shlezinger et al., 2012; Figure 1 and Table 1). In *Aspergillus nidulans*, asexual sporulation was associated with apoptotic-like cell death that included caspase activity (Thrane et al., 2004). In *Podospora anserina*, disruption of the two metacaspases (highly similar to caspases; Uren et al., 2000, see section Metacaspases) led to defects in sexual spore (ascospore) formation (Hamann et al., 2007). During ascus/ascospore maturation in *Coniochaete tetrasperma*, the number of ascospores per asci is reduced from 8 to 4 by means of a regulated cell death process (Raju and Perkins, 2000), which also occurs in *Neurospora* species and is controlled by the selfish genetic element known as Spore killer (*Sk*) (Raju, 1979; Turner and Perkins, 1979; Hammond et al., 2012). In *Coprinopsis cinereus*, cytological indicators of cell death were observed in immature basidiospores of meiotic mutants whose nuclei arrested after meiotic metaphase I (Lu et al., 2003) as well as prior to basidial differentiation in *Agaricus bisporus* (Umar and Griensven Van, 1997).

**Table 1:** PCD in filamentous fungi during differentiation or developmental processes.

**Stimulus**	**Organism**	**Genes**	**ROS**	**MC**	**References**
Heterokaryon incompatibility	*Neurospora crassa, Podospora anserina, Aspergillus niger*	*Nc*: HET-C1/2, PIN-C1/2, TOL, MAT-A/a, HET-6, UN-24, VIB-1, IME-2, HET-E *Pa*: HET-E, HET-S, NWD2, HELLP, SBP lipase	Yes	No	Saupe, 2000; Glass and Kaneko, 2003; Glass and Dementhon, 2006; Paoletti et al., 2007; Paoletti and Saupe, 2009; Hutchison et al., 2012; Zhao et al., 2015; Daskalov et al., 2016, 2017
Senescent cultures	*P. anserina, Botrytis cinerea*	*Pa*: Mitochondrial complex IV	Yes	Yes	Dufour et al., 2000; Osiewacz and Borghouts, 2000; Lorin et al., 2006; Osiewacz, 2011; Shlezinger et al., 2011b
Appressorium morphogenesis	*Magnaporthe grisea*	Several autophagy-related genes	NA	NA	Veneault-Fourrey et al., 2006; Liu et al., 2007; Kershaw and Talbot, 2009; Wilson and Talbot, 2009
Protoperithecia formation	*P. anserina*	*Pa*: PaATG1, PaATG8	NA	NA	Pinan-Lucarre et al., 2005
Asci maturation and ascospore formation	*Coniochaete tetrasperma, P. anserina*	–	NA	NA	Raju and Perkins, 2000; Hamann et al., 2007
Meiotic defects/Spore killer element	*Coprinopsis cinereus, N. sitophila, N. intermedia*	*Ns, Ni*: RSK	NA	NA	Raju, 1979; Turner and Perkins, 1979; Lu et al., 2003; Hammond et al., 2012
Basidial differentiation	*Agaricus bisporus*	–	NA	NA	Umar and Griensven Van, 1997
Conidiation	*A. nidulans*	*An*: PrpA	NA	Yes	Thrane et al., 2004

*Columns “ROS” and “MC” denote, for the effect of each stimulus, the involvement of reactive oxygen species (ROS) or metacaspases, respectively. NA, not assessed; Nc, N. crassa; Pa, P. anserina; Ns, N. sitophila; An, A. nidulans*.

### PCD during aging

*Podospora anserina* undergoes a senescence-type form of cell death characterized by a reorganization of the mitochondrial DNA, decrease in mycelium growth rate and increase in pigmentation and death of peripheral hyphae (Osiewacz, 2011; Bernhardt et al., 2014). *Podospora anserina* therefore has been used as a model for studying aging (Rizet, 1953). Senescence is associated with cytochrome *c* oxidase (complex IV) activity of the mitochondrial respiratory chain (Dufour et al., 2000; Osiewacz and Borghouts, 2000; Lorin et al., 2006). Although for many years the mitochondrial accumulation of ROS has been considered the key event underlying biological aging and lifespan in *P. anserina*, recent data indicates that other signals, including the mitochondrial quality control are also implicated in this process (Osiewacz and Bernhardt, 2013).

### PCD and non-self-recognition

PCD has also been associated with non-self-recognition in a number of filamentous fungi (Figure 1). Non-self-recognition in filamentous fungi is termed vegetative or heterokaryon incompatibility (HI) and has been described in both ascomycete and basidiomycete species (Rayner, 1996; Worrall, 1997; Saupe, 2000; Glass and Kaneko, 2003; Glass and Dementhon, 2006; Van der Nest et al., 2014). Non-self-recognition can occur at distance resulting in hyphal inhibition or as a consequence of hyphal or cell fusion between genetically different colonies, thus forming a heterokaryon where nuclei with different genetic backgrounds are present in the same cell. If nuclei within the heterokaryon are genetically dissimilar at the so called *het* loci, heterokaryotic parts of the colony are compartmentalized and cell death reactions are triggered, leading to a rejection of stable heterokaryon formation (Glass and Kaneko, 2003; Glass and Dementhon, 2006). HI has been shown to restrict viral transfer via hyphal fusion between genetically different fungal colonies and to prevent resource appropriation (Debets et al., 1994; Van Diepeningen et al., 1997; Debets and Griffiths, 1998; Biella et al., 2002).

When progeny or transformants have a duplication of incompatible *het* alleles or when incompatible heterokaryons are forced using auxotrophic markers, the heterokaryotic colony shows a macroscopic phenotype of slow growth and diminished conidiation, together with hyphal compartmentalization, lipid droplet accumulation, vacuolization and cell death (Perkins, 1975; Jacobson et al., 1998; Glass and Dementhon, 2006; Hutchison et al., 2009). HI is associated with the production of reactive oxygen species (ROS) and activation of phosphatidylinositol- and calcium (Ca^2+^)-related genes (Hutchison et al., 2009). Furthermore, nuclear DNA condensation and fragmentation, plasma membrane shrinkage, vesicle formation and internalization of vital dyes occur, drawing parallels between HI reactions and apoptosis in metazoans (Marek et al., 2003; Glass and Dementhon, 2006; Hutchison et al., 2009).

In spite of the genetic identification and characterization of various genes inducing HI in filamentous fungi, the molecular basis of the death reaction are still not well understood. A common feature among proteins inducing HI in filamentous fungi is the presence of a ~150 aa region termed the HET domain (PF06985, named after HETerokaryon incompatibility), suggesting that HI reactions induced by different HET domain proteins have similar downstream PCD pathways. This assumption is corroborated by the fact that the deletion of a single transcription factor, *vib-1* (vegetative incompatibility blocked-1), suppresses the HI reaction mediated by genetic differences at all molecularly characterized *het* loci in *Neurospora crassa* (Dementhon et al., 2006). Additionally, in *P. anserina*, autophagy was also associated with HI (Pinan-Lucarre et al., 2003, 2005). In support of this hypothesis, treatment with rapamycin, an inhibitor of TOR kinase, mimics the typical alterations of HI, namely the induction of *idi* (induced during incompatibility) genes and cytological alterations such as increased septation, vacuolization and coalescence of lipid droplets, suggesting that autophagic cell death is part of the mechanism of non-self-recognition-mediated PCD (Dementhon et al., 2003). However, inactivation of *idi-4* did not affect HI (Dementhon et al., 2004) and evidence suggests that fungal autophagy plays a protective role during some HI reactions (Pinan-Lucarre et al., 2003, 2005), similar to what has been observed in some plant models (Liu et al., 2005).

An example of a HI system in filamentous fungi for which the molecular mechanism of death is relatively well characterized is the *het-s/het-S* system of *P. anserina* (Saupe, 2011). In this HI system, the *het-s* allele encodes a prion protein (the HET-s protein) that can exist as a soluble monomer, in a state termed [Het-s^*^], or as infectious aggregates (a prion state), termed [Het-s]. Importantly, the [Het-s^*^] form can be transformed to the prion state when in contact with [Het-s]. Prion-infected [Het-s] strains trigger HI with [Het-S] strains, while strains containing the non-prion form, [Het-s^*^], do not. Cell death is induced when the HET-S protein is activated to form a pore-forming toxin upon interaction with the HET-s prion protein (Coustou et al., 1997; Daskalov et al., 2015, 2016), highlighting that this cell death process has molecular similarities with mammalian necroptosis (Daskalov et al., 2016, see below, in Fungal Nod-Like Receptors- Molecular Hubs for Cell Death Signaling and beyond?).

### PCD in response to stress

In addition to PCD associated with reproduction, aging and HI in fungi, the response to external stressors can also induce PCD. In fact, activation of PCD is a major treatment strategy for fungal infections in both animals and plants (Ramsdale, 2008). PCD can be triggered by incubating cells with cell death-inducing agents such as staurosporine, phytosphingosine, farnesol or acetic acid, as well as with UV light, oxidative stress and antifungal agents (Figure 1). Table 2 (and references therein) summarizes the wide array of stressors that induce PCD in filamentous fungi. It also denotes the different signaling and effector molecules that have been shown to be involved in the mode of action of each of the stressors. For more in-depth information on some of the signaling pathways, the readers are referred to the original publications. Cells treated with these stressors show typical apoptotic markers like DNA fragmentation, nuclear condensation, chromatin condensation and nuclear degradation as well as ROS accumulation and positive Annexin V and negative PI staining (Sharon et al., 2009). Increasing environmental stress can be associated with a shift from regulated toward uncontrolled death (Phillips et al., 2003).

**Table 2:** PCD in filamentous fungi exposed to cell death-inducing stimuli.

**Stimulus**	**Organism**	**Genes**	**Ca^2+^**	**ROS**	**MC**	**References**
**SPHINGOLIPIDS**						
Dihydrosphingosine	*Aspergillus nidulans*	–	NA	NA	NA	Cheng et al., 2003; Plesofsky et al., 2008
Phytosphingosine	*Neurospora crassa, A. nidulans*	*Nc*: Mitochondrial complex I subunits (NUO9.8, NUO14, NUO21, NUO21.3c, NUO30.4, NUO51, NUO78), AIF, AMID, ATP synthase subunit 4, aldehyde dehydrogenase, TRANSLIN, TRAX	No	Yes^c^	Yes^d^	Cheng et al., 2003; Castro et al., 2008; Plesofsky et al., 2008; Videira et al., 2009; Li et al., 2012; Fernandes et al., 2013
Ceramide	*N. crassa*	–	NA	NA	NA	Plesofsky et al., 2008
**CELL WALL OR PLASMA MEMBRANE-DISTURBING AGENTS**						
Amphotericin B	*Aspergillus fumigatus, N. crassa*	–	Yes	NA	No	Bowman et al., 2002; Mousavi and Robson, 2004; Munoz et al., 2014
Caspofungin	*A. fumigatus*	–	NA	NA	NA	Bowman et al., 2002
Itraconazole^a^	*A. fumigatus, Rhizopus oryzae, Cunninghamella bertholletiae, Mucor circinelloides*	–	NA	Yes	Yes	Shirazi and Kontoyiannis, 2013a,b
Posaconazole^a^	*R. oryzae, C. bertholletiae, M. circinelloides*	–	NA	Yes	Yes	Shirazi and Kontoyiannis, 2013a,b
Chitosan	*N. crassa, Fusarium eumartii*	*Nc*: NCU03639 (class 3 lipase), NCU04537 (monosaccharide trans-porter), NCU10521 (gluta-thione S-transferase-4)	Yes	NA	NA	Palma-Guerrero et al., 2009; Terrile et al., 2014; Lopez-Moya et al., 2016
**PLANT-DERIVED COMPOUNDS**						
Camalexin	*Botrytis cinerea*	*Bc*: BcBir1	NA	Yes	NA	Shlezinger et al., 2011b
Hexanoic (caproic) acid	*B. cinerea, Ustilago maydis*	*Bc*: BcNma; *Um*: Mfe2, Mfe2b, Had1, Had2	NA	NA	NA	Finkelshtein et al., 2011; Kretschmer et al., 2012
α-Tomatine	*Fusarium oxysporum*	–	Yes^e^	Yes	Yes	Ito et al., 2007
Sugarwin2	*Colletotrichum falcatum*	–	NA	NA	NA	Franco et al., 2014
Dill oil	*Aspergillus flavus*	–	NA	Yes	NA	Tian et al., 2012
Anethole	*A. fumigatus*	–	NA	Yes	Yes	Fujita et al., 2014
Defensins	*N. crassa*	*Nc*: GCS	Yes	NA	NA	Munoz et al., 2014
Anacardic acid	*Magnaporthe oryzae*		NA	No^f^	No	Muzaffar et al., 2016
Perillaldehyde	*Aspergillus flavus*	–	Yes	Yes	Yes	Tian et al., 2016
**FUNGAL- AND BACTERIAL-DERIVED COMPOUNDS**						
PAF	*N. crassa, A. nidulans*	*An*: FadA	Yes	Yes	NA	Leiter et al., 2005; Binder et al., 2010
Trichokonin VI	*F. oxysporum, Ascochyta citrullina, B. cinerea, Phytophtora parasitica, Verticillium dahlia*	–	Yes^e^	No	No	Shi et al., 2012
Ophiobolin A	*M. circinelloides, Rhizopus stolonifer*	–	NA	NA	NA	Krizsan et al., 2010
L-amino acid oxidase	*B. cinerea*	–	NA	Yes	NA	Cheng et al., 2012
Lovastatin	*Mucor racemosus, B. cinerea*	*Mra*: MRas1, MRas3, (cAMP signaling pathway), PI3K	NA	NA	NA	Roze and Linz, 1998; Shlezinger et al., 2011b
Farnesol	*A. nidulans, Fusarium graminearum, Penicillium expansum, Metarhizium robertsii, A. flavus*	*An*: PrpA, NucA, CycA, FadA, SfaD, AifA, PkcA, AtgH, PkcA, CasA, CasB, HacA, MAPK signaling pathway *Mro*: MrBI-1	NA	Yes	Yes	Semighini et al., 2006a,b, 2008; Savoldi et al., 2008; Colabardini et al., 2010; Dinamarco et al., 2010; Liu et al., 2010; de Castro Pimentel Figueiredo et al., 2011; Wang et al., 2014; Chen et al., 2015
Staurosporine	*N. crassa*	*Nc*: Mitochondrial complex I subunits (NUO9.8, NUO14, NUO30.4, NUO51), NDE-1, ABC-3, CZT-1, TAH-3, CAT-1, AMID-2	Yes	Yes	No	Castro et al., 2010; Fernandes et al., 2011, 2013; Goncalves et al., 2014a,b; Gonçalves et al., 2014; Goncalves et al., 2015
WH1 fungin	*Rhizoctonia solani*	–	NA	Yes	Yes	Qi et al., 2010
***De novo*** **DESIGNED ANTIMICROBIAL PEPTIDES**						
PAF26, PAF95, PAF96	*N. crassa, A. fumigatus*	–	Yes	NA	NA	Munoz et al., 2012, 2013
_D_(KLAKLAK)_2_	*R. oryzae, M. circinelloides*	–	NA	Yes	Yes	Barbu et al., 2013
**OXIDATIVE STRESS INDUCERS**						
Hydrogen peroxide	*A. fumigatus, B. cinerea, Colletotrichum trifolii, N. crassa*	*Bc*: BcBir1, BcNma	Yes	Yes	No	Mousavi and Robson, 2004; Chen and Dickman, 2005; Castro et al., 2008; Videira et al., 2009; Finkelshtein et al., 2011; Shlezinger et al., 2011b; Carneiro et al., 2012b; Duarte and Videira, 2012; Munoz et al., 2015
Paraquat	*N. crassa, Podospora anserina*	*Nc*: NDE-1, NDE-2^b^	NA	Yes	NA	Duarte and Videira, 2009; Carneiro et al., 2012a; Wiemer and Osiewacz, 2014
**METALS**						
Cu^2+^	*Heliscus submersus, Flagellospora curta, Varicosporium elodeae*	–	NA	Yes	Yes	Azevedo et al., 2009
Zn^2+^	*Heliscus submersus, Flagellospora curta, Varicosporium elodeae, Fusarium verticillioides*	–	NA	Yes	Yes	Azevedo et al., 2009; Savi et al., 2013
Cr (VI)	*N. crassa*	–	NA	Yes	NA	Gaddameedi et al., 2011
**OSMOTIC STRESS**						
Water (7 days)	*B. cinerea*	*Bc*: BcNma	NA	NA	NA	Finkelshtein et al., 2011
Salt (NaCl)	*Fusarium proliferatum*	*Fp*: FpHog1	NA	Yes	NA	Adam et al., 2008
Hyperosmotic stress	*F. proliferatum*	*Fp*: FpHog1	NA	Yes	NA	Adam et al., 2008
**PHOTO-INDUCTION**						
Ultraviolet light	*C. trifolii*	–	NA	Yes	NA	Chen and Dickman, 2005
Photodynamic inhibition (aPI)	*Trichophyton rubrum*	–	NA	Yes	NA	Baltazar Lde et al., 2013
**NUTRIENT LIMITATION**						
Carbon starvation	*A. fumigatus, A. nidulans, Aspergillus niger*	*An*: AtmA, XprG, NagA, HxkC	NA	Yes	Yes	Mousavi and Robson, 2003; Emri et al., 2005; Richie et al., 2007; Nitsche et al., 2012; Krohn et al., 2014;Katz et al., 2016
Iron starvation	*R. oryzae*	–	NA	Yes	Yes	Shirazi et al., 2015
Auxotrophic strains	*N. crassa*	–	NA	Yes	NA	Strauss, 1958; Munkres, 1976
**GENETIC INDUCTION**						
Dominant activated Ras	*Colletotrichum trifolii*	*Ct*: CtRas	NA	Yes	NA	Chen and Dickman, 2005
Δ*pig-a*; *pig-a* conditional mutant	*A. fumigatus*	*Af*: PigA, QutG, PI3K	Yes	NA	No	Li et al., 2007; Yan et al., 2013
Δ*choC*	*A. nidulans*	*An*: ChoC	NA	NA	NA	Tao et al., 2010)
Kalilo plasmid-bearing strains	*N. crassa, Neurospora intermedia*	–	NA	NA	NA	Griffiths et al., 1984; Bok et al., 2003
Human BAX expression	*Colletotrichum gloeosporioides*	–	NA	NA	NA	Barhoom and Sharon, 2007
**OTHERS**						
Heat (42°C)	*Pleurotus ostreatus, Pleurotus pulmonarius*	–	NA	Yes	NA	Song et al., 2014
Moderate heat shock (45°C) + glucose deprivation (2-deoxyglucose)	*N. crassa*	*Nc*: CEL-1, OS-2	NA	NA	NA	Plesofsky-Vig and Brambl, 1995; Plesofsky et al., 2008
Confrontation assays	*P. anserina, Penicillium chrysogenum*	*Pa*: PaNOX1, PaASK1	NA	Yes	NA	Silar, 2005

a *In combination with tacrolimus, antimycin A or benzohydroxamate (Shirazi and Kontoyiannis, 2013a,b)*.

b *A double mutant strain Δnde-1Δnde-2 is substantially more resistant to paraquat than wild type (Carneiro et al., 2012b)*.

c *Phytosphingosine induces reactive oxygen species (ROS) production although ROS scavenging does not block cell death*.

d *Only ΔcasA was tested and A. nidulans also possesses casB*.

e *Although alterations in the intracellular levels of Ca^2+^ were not measured, inhibition of Ca^2+^ channels or addition of Ca^2+^ chelators blocks cell death (Ito et al., 2007; Shi et al., 2012)*.

f *Treatment with anacardic acid caused a decrease in ROS accumulation instead of the common increase in ROS observed for other compounds (Muzaffar et al., 2016). Columns “Ca^2+^,” “ROS,” and “MC” denote, for the effect of each stimulus, the involvement of Ca^2+^, ROS or metacaspases, respectively. NA, not assessed. Nc, N. crassa; Bc, B. cinerea; Mra, M. racemosus; Um, U. maydis; An, A. nidulans; Mro, M. robertsii; Fp, F. proliferatum; Ct, C. trifolii; Af, A. fumigatus; Pa, P. anserina*.

### PCD during plant infection

Fungal PCD has also been implicated in plant infection (Figure 1). For example, in the rice blast pathogen *Magnaporthe grisea*, autophagic cell death of conidia is essential for infection via a specialized type of cell (appressorium), which is required to penetrate the outer cuticle of leaves and stems of rice plants (Wilson and Talbot, 2009). Appressorium morphogenesis requires a mitotic event, nuclear migration, and death of the asexual spore that initiates the infection (Veneault-Fourrey et al., 2006). Conidial death is mediated by autophagy since disruption of the autophagy-mediator gene *MgATG8* prevents cell death (Liu et al., 2007; Kershaw and Talbot, 2009); the *MgATG8* mutant forms appressoria, but is unable to penetrate plant tissues and cause disease.

Cell death has also been observed in necrotrophic fungi in response to the host defense machinery during plant infection. For example, *Arabidopsis* cells produce a phytoalexin called camalexin that induces cell death in the necrotrophic fungus *Botrytis cinerea* (Shlezinger et al., 2011b). Overexpression of the anti-apoptotic gene *BcBIR1* in *B. cinerea* confers enhanced pathogenicity and resistance to cell death (Shlezinger et al., 2011b), while a Δ*BcBIR1* mutant exhibits the opposite phenotype, i.e., hypersensitivity to cell death and reduced virulence. Another plant defense compound, hexanoic acid, induces cell death in *B. cinerea* through BcNma, the orthologue of the mammalian pro-apoptotic protein HtrA2 (Roze and Linz, 1998; Finkelshtein et al., 2011; Shlezinger et al., 2011b). Tomato cells produce the saponin α-tomatine, a sesquiterpene glycoside with fungicidal activity that induces ROS- and metacaspase-dependent cell death in *Fusarium oxysporum* (Ito et al., 2007). In addition, during host-pathogen interactions, fungal cells may manipulate the mechanisms of plant PCD for their benefit. For example, *Alternaria alternata* produces a mycotoxin called AAL, with structural similarities to sphinganine, that induces cell death in tomato cells by interfering with the sphingolipid biosynthesis (Brandwagt et al., 2000), leading to the accumulation of dihydrosphingosine and cell death.

## Molecular mediators of regulated cell death

### Calcium (Ca^2+^), the mitochondrial electron transport chain and ROS

**Figure 2:**
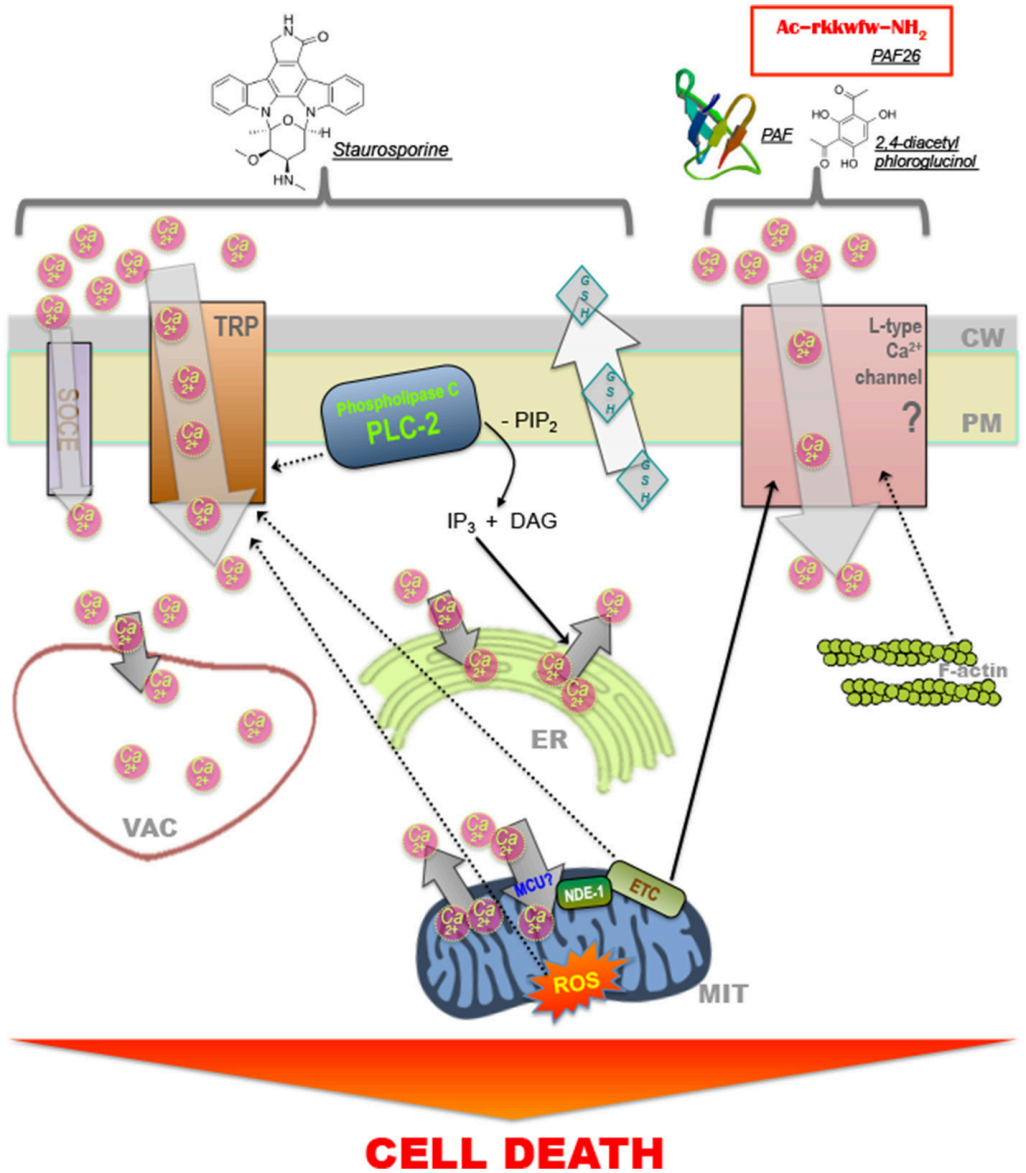
Model for Ca^2+^ transport and signaling involved in cell death in filamentous fungi. Model for staurosporine induced modifications in intracellular Ca^2+^ dynamics that precede induction of cell death in *N. crassa* (Goncalves et al., 2014a). The phospholipase C-family protein PLC-2 plays a pivotal role during the process, which occurs via the recruitment of Ca^2+^ from the extracellular space, through a TRP-like channel via store-operated Ca^2+^ entry (SOCE) and from internal stores such as the endoplasmic reticulum (via a IP_3_-activated channel) or the mitochondria; ROS formation is necessary for the recruitment of Ca^2+^ to the cytosol (Goncalves et al., 2015). Vacuoles, other acidic vesicles, the endoplasmic reticulum and mitochondria uptake Ca^2+^ to buffer the concentration of ions in the cytosol. The antifungal peptides Penicillium AntiFungal (Binder et al., 2010) and PAF26 (Munoz et al., 2012) and 2,4- diacetylphloroglucinol (Troppens et al., 2013) also induce cell death and the recruitment of Ca^2+^ to the cytosol. In this case, Ca^2+^ recruitment occurs, at least partially, through L-type Ca^2+^ channels, which are affected by the stability of the intracellular F-actin network. The mitochondrial electron transport chain (ETC) regulates increases in intracellular Ca^2+^ during cell death (Munoz et al., 2012; Goncalves et al., 2015). Dashed arrows indicate that a relationship between the two elements has been suggested but not fully proven.

Among the different molecules and pathways identified as playing a role during fungal cell death, Ca^2+^-triggered signaling seems to be frequently involved in responding to treatment with different compounds (Figure 2). Transient or stable modifications in the cytosolic levels of Ca^2+^ can operate both as pro-survival or pro-death signals. To regulate the intracellular concentration of Ca^2+^, cells chelate, compartmentalize or remove the ion using active (pumps and transporters) and passive (Ca^2+^-binding proteins) systems (Berridge et al., 2000; Carafoli, 2002; Clapham, 2007; Cerella et al., 2010). In *S. cerevisiae*, the elevation of cytosolic Ca^2+^ levels is a common response to antifungal agents, including the plant essential oils carvacrol (Rao et al., 2010) and eugenol (Roberts et al., 2012), amiodarone (Gupta et al., 2003; Pozniakovsky et al., 2005) and ER stress-inducing agents like tunicamycin and azole drugs (Bonilla et al., 2002; Martin et al., 2011). In *N. crassa*, a rise in the cytosolic levels of Ca^2+^ was also associated with treatment with chitosan (Palma-Guerrero et al., 2009), antifungal proteins PAF (Binder et al., 2010), and PAF26 (Munoz et al., 2012), 2,4-diacetylphloroglucinol (Troppens et al., 2013), plant defensins (Munoz et al., 2014), and staurosporine (Goncalves et al., 2014a, 2015). Staurosporine is a natural alkaloid that has traditionally been used to inhibit protein kinases, and which induce apoptosis in mammalian cells through both caspase-dependent and caspase-independent mechanisms (Belmokhtar et al., 2001). Phospholipase C seems to be a pivotal player coordinating recruitment of Ca^2+^ to the cytosol during staurosporine-induced cell death as cells lacking the phospolipase C gene *plc-2* exhibit increased survival and a staurosporine-induced cytosolic Ca^2+^ signature is abolished (Goncalves et al., 2014a). The importance of extracellular Ca^2+^ during staurosporine-induced fungal PCD is supported by the observation that cell death in *N. crassa* is exacerbated in Ca^2+^-free medium and inhibited when excess Ca^2+^ is present (Gonçalves et al., 2014). Consistent with these observations, a similar protection from fungal PCD was observed by the presence of an excessive amount of extracellular Ca^2+^ in occidiofungin-treated *S. cerevisiae* cells and chitosan-treated *N. crassa* cells (Lopez-Moya et al., 2016; Robinson et al., 2017).

The mitochondrial respiratory chain of *N. crassa* is involved in the regulation of intracellular Ca^2+^ dynamics upon treatment with staurosporine (Goncalves and Videira, 2015; Goncalves et al., 2015). More specifically, deletion of certain subunits of complex I, namely NUO51 and NUO14, disrupts Ca^2+^ signaling after treatment with staurosporine and results in hypersensitivity to the drug (Castro et al., 2010). Chemical disruption of other different components of the mitochondrial respiratory chain also led to defective Ca^2+^ responses during staurosporine-induced cell death (Goncalves et al., 2015).

In addition to changes in Ca^2+^ levels, the export of GSH and the accumulation of ROS are determinant events during fungal cell death. For example, ROS accumulation has been shown to occur after treatment with staurosporine (Castro et al., 2010), azole drugs (Shirazi and Kontoyiannis, 2013a,b), PAF (Leiter et al., 2005), farnesol (Semighini et al., 2006a; Liu et al., 2010), and during HI (Hutchison et al., 2009). In addition, *N. crassa* cells, like higher eukaryotes, export GSH which seems to be a cause rather than a consequence during the execution of staurosporine- and phytosphingosine-induced cell death (Fernandes et al., 2013). This phenomenon leads to an imbalance of intracellular GSH/GSSG ratio that favors the accumulation of ROS and the oxidation of cellular components. Importantly, staurosporine-induced cell death is prevented by the supplementation of exogenous GSH or its precursor N-acetyl-cysteine (NAC) (Castro et al., 2010). Interestingly, addition of exogenous GSH or NAC blocked the staurosporine-induced intracellular Ca^2+^ signature, indicating that it is dependent on ROS (Goncalves et al., 2015).

### The BCL-2 family of proteins and other members of the intrinsic cell death pathway

Most of the common regulators and executioners of mammalian apoptosis do not have evident homologs in fungal genomes (Sharon et al., 2009; Shlezinger et al., 2012). However, it is clear that fungi undergo an apoptotic-like cell death in response to several stimuli, suggesting that proteins with low (or no) sequence similarity might behave as functional analogs of the mammalian mediators of cell death. In line with this, heterologous expression of mammalian BCL-2 family members in fungi can induce or prevent cell death (Sato et al., 1994; Longo et al., 1997; Xu and Reed, 1998; Polcic and Forte, 2003; Barhoom and Sharon, 2007). Filamentous fungi possess more proteins with homology to cell death-related proteins of mammalian cells as compared to *S. cerevisiae* (Fedorova et al., 2005). Moreover, the similarity between some proteins involved in cell death in humans and filamentous fungi is higher than the similarity of these same proteins between yeast and filamentous fungal species (Fedorova et al., 2005). In addition to the fact that many filamentous fungi have been extensively used as model organisms for cell biology, these two features make them especially attractive to study PCD. Sequence conservation between mammalian and fungal cell death mediators is normally domain-centered, with conservation of elements important for an apoptotic role and divergence of the remainder of the protein sequence (Sharon et al., 2009; Shlezinger et al., 2012).

Using an *in silico* domain-based procedure, Shlezinger et al. identified putative fungal mediators of cell death including the BIR (baculovirus inhibitor of apoptosis protein repeat) domain (Shlezinger et al., 2011a). One BIR-containing protein was identified in *B. cinerea* (BcBir1) and described as an anti-apoptotic molecule involved in interactions with host plants (Shlezinger et al., 2011b). A pro-apoptotic protein called Bxi1/Ybh3 containing a BH3-like signature at the C-terminal portion, triggered PCD in *S. cerevisiae* (Buttner et al., 2011). BH3 domains are present in some members of the BCL-2 family (Chipuk et al., 2010). Homologues of Bxi1 in other fungi were predicted but functional verification has not been tested. Recently, an orthologue of the mammalian Bax inhibitor 1, MrBI-1 in *Metarhizium robertsii* was shown to be involved in the anti-death response of this fungal species to farnesol (Chen et al., 2015).

The mitochondrial component of mammalian apoptosis (classically termed the *intrinsic pathway*) is more conserved in fungi. Cytochrome *c* functions as an electron carrier of the mitochondrial inter-membrane space, but becomes a pro-apoptogenic molecule upon induction of cell death (Tait and Green, 2010). In *S. cerevisiae*, cytochrome *c* (Cyc1) is released from mitochondria upon exposure to acetic acid (Ludovico et al., 2002; Pereira et al., 2007). In filamentous fungal species, this phenomenon was shown to occur in response to treatment with the anti-fungal peptides _D_(KLAKLAK)_2_ (Barbu et al., 2013) and WH1 fungin (Qi et al., 2010), as well as upon exposure to a combined treatment of azole drugs and tacrolimus (Shirazi and Kontoyiannis, 2013a).

In mammals, AIF and EndoG are DNA nucleases that normally reside in the mitochondria where they play a role in the maintenance of the respiratory chain (Vahsen et al., 2004) and in mitochondrial DNA replication (Cote and Ruiz-Carrillo, 1993), respectively. In mammalian cells, upon induction of apoptosis, these two molecules translocate to the nucleus and cleave DNA (Tait and Green, 2010). In yeast, the AIF homolog, Aif1, also migrates to the nucleus in response to a variety of stresses where it causes DNA condensation and fragmentation (Wissing et al., 2004). In *N. crassa, aif* disruption leads to increased resistance to phytosphingosine and hydrogen peroxide (Castro et al., 2008). In *A. nidulans, aifA* is induced by farnesol and deletion of *aifA* confers increased sensitivity to this drug and hydrogen peroxide (Savoldi et al., 2008; Dinamarco et al., 2010). These apparently contrasting results might be linked to compensatory mechanisms since AIF belongs to a small family of proteins that also includes the AIF-homologous mitochondrion-associated inducers of death (AMIDs). Human AMID (or PRG3) is a NAD(P)H oxidase that induces caspase-independent apoptosis (Wu et al., 2002). In *N. crassa*, the AIF-family is composed of AIF, AMID, and AMID-2 (Carneiro et al., 2012a). AIF and AMID proteins are related to the alternative NAD(P)H dehydrogenases present in the mitochondria of fungi, plants, protists and bacteria and are particularly relevant because they oxidize NAD(P)H, reduce quinones and serve as entry points for electrons into the respiratory chain (Videira and Duarte, 2002; Carneiro et al., 2012a). Disruption of *N. crassa* AMID results in increased sensitivity to phytosphingosine and hydrogen peroxide (Castro et al., 2008), while single or multiple deletion of the different alternative NAD(P)H dehydrogenases results in abnormal accumulation of ROS and altered sensitivity to cell death stimuli (Goncalves and Videira, 2015). In *P. anserina*, deletion of *amid, amid2*, and *aif2* results in an increase in lifespan (Hamann et al., 2007; Brust et al., 2010).

The mammalian EndoG seems to be involved in mitochondrial DNA replication (Cote and Ruiz-Carrillo, 1993), but is released from the mitochondria in a caspase-independent manner and cleaves nuclear DNA during apoptosis (Tait and Green, 2010). In yeast, Nuc1 seems to behave similarly to its human EndoG homolog, as it translocates to the nucleus after exposure of yeast cells to hydrogen peroxide (Burhans and Weinberger, 2007). However, in *A. nidulans*, the EndoG homolog NucA is not involved in farnesol-induced cell death (de Castro Pimentel Figueiredo et al., 2011). In animals, HtrA2/Omi is a mitochondrial pro-apoptotic protein that binds to inhibitor of apoptosis proteins and alleviates their inhibitory effect on caspases (Tait and Green, 2010). In yeast, its homolog Nma111, although not mitochondrial, but rather nucleus-localized, is also pro-apoptotic (Fahrenkrog et al., 2004). In *B. cinerea*, genetic manipulation of the HtrA2/Omi homolog BcNma by overexpression or deletion leads to enhanced or reduced appearance of apoptotic markers, respectively (Finkelshtein et al., 2011). However, the overexpression and mutant strains responded similarly to wild type cells when exposed to different cell death-inducing stresses.

### Metacaspases

In animals, caspases are synthesized as inactive zymogens (procaspases) and are activated upon a cell death stimulus. Based on their structure and function, caspases can be classified in three groups: inflammatory, initiator and effector caspases (Fuentes-Prior and Salvesen, 2004; Pop and Salvesen, 2009). Two caspase-related families have also been identified: the metacaspases and the paracaspases (Uren et al., 2000). Metacaspases (as well as paracaspases) have the catalytic histidine/cysteine dyad and secondary structure predictions indicate the presence of the caspase/hemoglobinase fold (Uren et al., 2000; Vercammen et al., 2007). Metacaspases possess sequence similarity with mammalian caspases, in particular in the catalytic p20 and p10 domains. However, metacaspases have a major difference with caspases: instead of cleaving after aspartic acid residues, they cleave after arginine or lysine residues (Vercammen et al., 2004, 2006; Watanabe and Lam, 2005). In humans, paracaspases are involved in the development of MALT lymphoma, but apparently not in cell death execution (Uren et al., 2000). Recently, it has been suggested that paracaspases are a subclass of metacaspases (Hulpiau et al., 2016). Metacaspases have been traditionally divided in two types (I and II). Type II metacaspases are found only in plants, while the distribution of type I metacaspases is more widespread across eukaryotes (Carmona-Gutierrez et al., 2010b). Type I metacaspases possess an N′-terminal region evocative of the pro-domain of initiator and inflammatory caspases with a proline-rich repeat motif (Uren et al., 2000). Type II metacaspases lack such a pro-domain (Uren et al., 2000; Vercammen et al., 2004). Recently, a third type of metacaspase, type III, containing a rearrangement of domain structures between N- and C-terminus has been suggested in phytoplanktonic protists (Choi and Berges, 2013).

Although differing opinions regarding the nomenclature and function of metacaspases occur (Vercammen et al., 2007; Carmona-Gutierrez et al., 2010b; Enoksson and Salvesen, 2010), more recent evidence points to a clear role during cell death. In plants, the conserved Tudor Staphylococcal Nuclease (TSN) was described as a natural substrate of the type II metacaspase of *Picea abies* mcII-Pa (Sundstrom et al., 2009); mcII-Pa activity on TSN is associated with cell death during stress and development. The *Arabidopsis thaliana* Type I metacaspases AtMC1 and AtMC2 antagonistically control PCD in response to pathogen attack (Coll et al., 2010).

In *S. cerevisiae*, the sole metacaspase Yca1 (also described as Mca1) is cleaved like a typical caspase upon treatment with hydrogen peroxide, demonstrating its role as pro-death molecule (Madeo et al., 2002). However, recent work has shown that Yca1 can play a cytoprotective role during removal of misfolded proteins, which lead to yeast life span extension (Hill et al., 2014; Liu, 2014). In the filamentous fungus *Aspergillus fumigatus*, apoptotic-like cell death occurring after exhaustion of the carbon source and entry into the stationary phase of growth is associated with intracellular activity against caspase-1 and -8 substrates and is blocked by the pan-caspase inhibitor Z-VAD-fmk (Mousavi and Robson, 2003). However, the two metacaspases identified in the *A. fumigatus* genome (CasA and CasB) do not participate in the response to hydrogen peroxide, amphotericin B or phytosphingosine-induced cell death (Cheng et al., 2003; Mousavi and Robson, 2004) as a Δ*casA* Δ*casB* mutant showed a similar sensitivity to pro-apoptotic stimuli as a wild type strain (Richie et al., 2007). In *P. anserina*, disruption of either or both of the predicted metacaspases (PaMCA1 and PaMCA2) led to an increase in lifespan, particularly the Δ*PaMca1* mutant (Hamann et al., 2007). Metacaspase-dependent activity occurred in senescent but not in juvenile cultures, while 15 day-old mycelia from the Δ*PaMca1* mutant was more resistant to hydrogen peroxide. In *N. crassa*, the two predicted metacaspases (MCA-1A and MCA-1B) are not required for HI-related cell death (Hutchison et al., 2009), although transcripts for both metacaspases increased during HI and *mca-1A* is overexpressed in response to phytosphingosine (Videira et al., 2009). Metacaspases have also been linked to the response of other filamentous fungi to a number of stimuli (Table 2).

PARP-1 (poly ADP-ribose polymerase) is involved in DNA repair and is a target of caspases during mammalian apoptosis (Dantzer et al., 2000; Tait and Green, 2010). PARP homologs are absent in yeast but are present in filamentous fungi (Fedorova et al., 2005). The *A. nidulans* PARP homolog PrpA plays a role in the response to DNA damage and is required for farnesol-induced cell death (Semighini et al., 2006b). In *A. nidulans*, metacaspases have been implicated in degradation of a PARP-like protein in cell death associated with asexual sporulation processes (Thrane et al., 2004). PARP was also described as a substrate of *P. anserina* metacaspases (Strobel and Osiewacz, 2013).

### Fungal NOD-like receptors–molecular hubs for cell death signaling and beyond?

**Figure 3:**
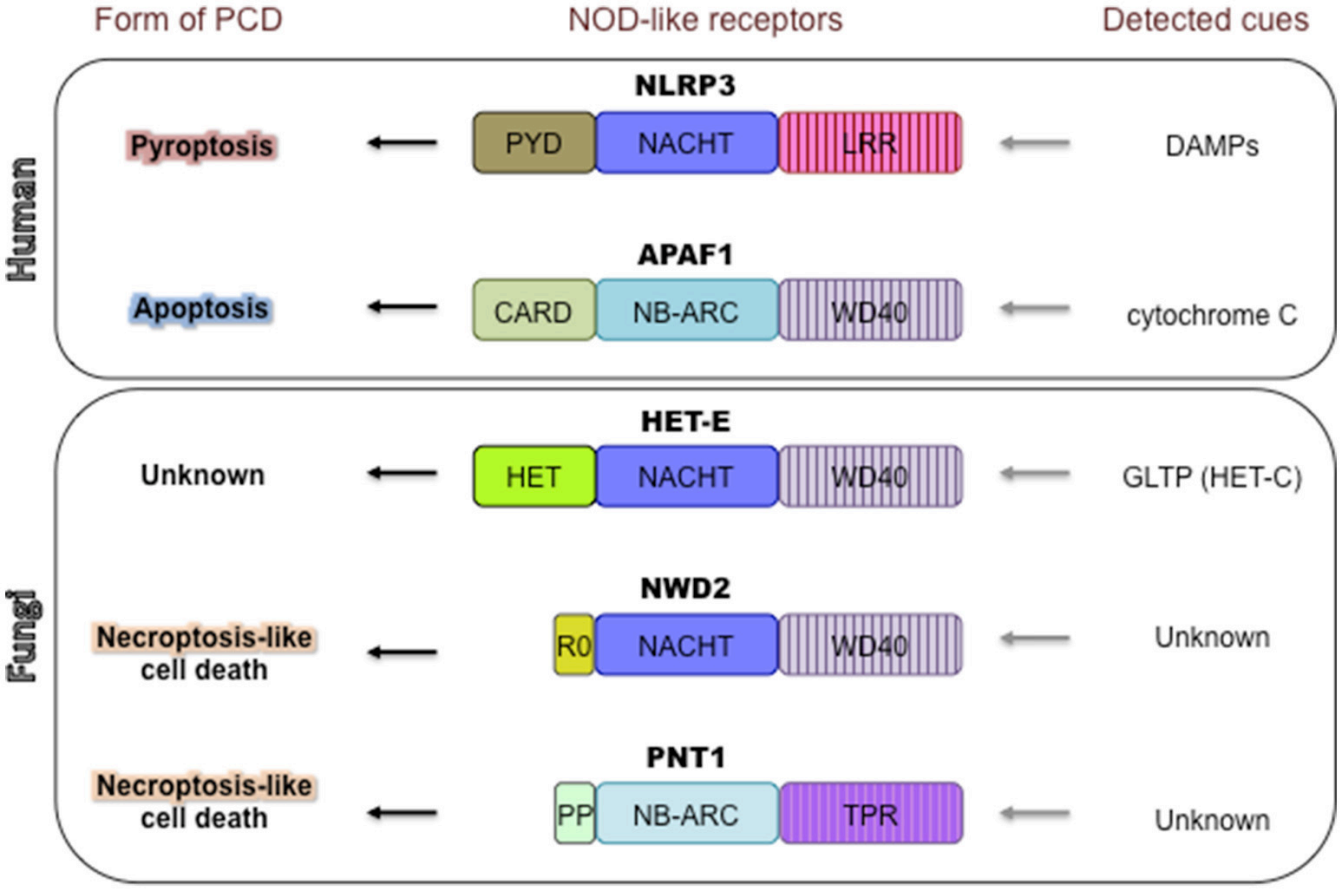
NOD-like receptors control various forms of PCD in mammals and filamentous fungi. NLRP3 and APAF1, given as examples, control pyroptosis and apoptosis, respectively. NLRP3 activity is triggered by various DAMPs (damage-associated molecular patterns), which leads to highly inflammatory pyroptotic cell death (Jones et al., 2016). APAF1 controls the intrinsic apoptotic cell death pathway in mammals (Shakeri et al., 2017). APAF1 is activated by cytochrome C, released from damaged mitochondria. Three examples are given of fungal NOD-like receptors controlling PCD. HET-E controls the HI PCD reaction in the ascomycete *P. anserina*. PCD is triggered by incompatible allelic variants of HET-C, a glycolipid transfer protein (GLTP). The downstream signaling pathway of HET-E activation and specific mechanism of cell death are currently unclear. Some fungal NLRs (e.g., NWD2 of *P. anserina* or PNT1 of *Chaetomium globosum*) use short amyloid motifs (R0 or PP (pseudo-palindromic) respectively) to induce a cell death reaction that has been linked to *necroptosis*—inflammatory PCD in metazoans (Loquet and Saupe, 2017). The molecular cues and events activating NWD2 and PNT1 are not yet identified.

Recently, several PCD-controlling molecular players in fungi have been identified that are analogous to NLR proteins [Nucleotide-Binding Domain (NBD) and Leucine-Rich Repeats (LRRs)], also termed NOD-like receptors (Figure 3), which are major constituents of innate immune defenses in plants and metazoans (Proell et al., 2008; Duxbury et al., 2016; Jones et al., 2016; Kim et al., 2016). In plants, NLRs [or R (resistance) proteins] control a characteristic cell death reaction, termed the *hypersensitive response*, which prevents the spread of pathogenic agents inside plant tissues (Jones and Dangl, 2006). In metazoans, the activity of some NLRs can trigger apoptosis or lead to a highly inflammatory form of PCD known as *pyroptosis* (Da Silva Correia et al., 2007; Larock and Cookson, 2013; Jones et al., 2016). NLR proteins are intracellular, multi-domain molecular sensors, detecting endogenous or exogenous danger signals or pathogen (or microbial)-associated molecular patterns (PAMPs or MAMPs) (Proell et al., 2008). Most NLR proteins have a modular domain organization with a central NBD domain, an N-terminal effector domain—crucial for downstream signaling—and a C-terminal LRR-containing domain (Jones et al., 2016). Two different types of NBD domains can be encountered; the NB-ARC type (Nucleotide-Binding Domain shared by APAF-1, R proteins and CED-4) mostly present in plants, and the NACHT type (shared by the apoptosis inhibitory protein NAIP and the proteins CIITA, HET-E and TP1), which are predominant in metazoans (Jones et al., 2016). In the current paradigm for NLR functioning, the NLR sensors undergo an oligomerization process mediated by the NACHT/NB-ARC domains resulting in a transition from inactive to active state. The formation of a multimeric signaling platform results in proximity-induced activation of the N-terminal domains, which directly or indirectly induce specific molecular responses (Danot et al., 2009). The similar architectures of plant and metazoan NLRs is believed to be the result of evolutionary convergence rather than orthology (Urbach and Ausubel, 2017).

In fungi, more than 5600 putative NLR-like genes (“NLR-like” is used to signify architectures not containing LRRs) have been bioinformatically identified in 198 genomes, corresponding to 164 different species (Dyrka et al., 2014). NLR-like genes are present in all branches of the fungal kingdom with the exception of yeasts (Dyrka et al., 2014). The majority of fungal NLR-like genes encode a NACHT type of NBD, although NB-ARC domains are also present (1:5 ratio with NACHT). Three different types of super-structure-forming repeats (ANK, TPR, or WD40) are most often found at the C-terminus of fungal NLRs. Some of these C-terminal repeat-containing domains define the signal specificity of the receptors and possibly, as suspected for plant and metazoan NLRs, negatively regulate the NBD-mediated oligomerization process (Chevanne et al., 2010; Daskalov et al., 2015). For various fungal NLR-like proteins, intraspecific variations in repeat number have been reported (Dyrka et al., 2014) and positive diversifying selection on codons within the WD40 repeat region of NLR-like HI-inducing proteins HET-E, HET-D, HET-R have been identified (Paoletti et al., 2007). The process of inter- and intragenic repeat shuffling and positive diversifying selection acting on sequence level have been proposed to lead to an extremely diverse and vast non-self-recognition repertoire for this class of receptors (Paoletti et al., 2007; Chevanne et al., 2010).

The N-terminal effector domains of NLR proteins often serve to recruit additional molecular players important for signaling (Vajjhala et al., 2017). In metazoan NLRs, some N-terminal domains initiate the formation of high-order filaments termed “specks” of an adapter protein called ASC, which leads to cell death through the activation of various caspases (Hoss et al., 2017; Vajjhala et al., 2017). The nucleated-polymerization principle (or prion-like principle) of activation described for ASC is proposed to have broader implications in metazoan innate-immunity (Cai et al., 2014). The fungal NLR-like proteins bear an impressive variety of N-terminal domains with at least 12 different described classes, some of which encode putative lipases, phosphorylases and pore-forming toxin activities (Dyrka et al., 2014). One frequently found effector domain in the fungal NLR-like proteins is the HET domain (for example, in HET-E, HET-D, and HET-R) (Paoletti and Clave, 2007). The HET domain is instrumental for the HI PCD reaction, but its molecular function is currently unknown. However, it has been reported to show similarity to an effector domain termed TIR (Toll/interleukin-1 receptor) identified in various plant and metazoan innate immunity proteins (Paoletti and Clave, 2007; Dyrka et al., 2014; Duxbury et al., 2016). TIR domains function as adaptor domains mediating homotypic protein-protein interactions initiating PCD-associated signaling cascades (Ve et al., 2015).

In some instances, genes that encode for NLR effector domains lack any other regulatory motif (Daskalov et al., 2012; Loquet and Saupe, 2017). In this case, the effector activity can be controlled by an NLR protein encoded by an adjacent gene (Daskalov et al., 2015). The interaction between the NLR and the effector protein is dependent on a short amyloid-forming motif/domain with prion-like features (Daskalov et al., 2015; Loquet and Saupe, 2017) that serves to transduce the signal from the activated NLR toward the effector (Figure 3) (Saupe et al., 2016). Such NLR/effector functional units using amyloid-based signal transduction coexist with “all-in-one” architectures (where the effector is an integral part of the NLR) in a single fungal genome. Furthermore, some of the signaling amyloids have diversified into distinct families that share a specific primary sequence pattern while others appear to have acquired the signaling role in a convergent manner (Daskalov et al., 2016; Loquet and Saupe, 2017).

The NLR-based amyloid signal transduction has been evolutionarily linked to a form of PCD in metazoans termed *necroptosis* (Daskalov et al., 2016; Grootjans et al., 2017). Necroptotic cell death relies on the protein kinases RIP1 and RIP3, which form amyloid-based signaling complexes mediated by a short (~19aa) motif termed RHIM (RIP homotypic interaction motif) (Rebsamen et al., 2009; Orozco et al., 2014). The RHIM motif seems to be evolutionary related to some of the signaling amyloids attached to the fungal NLR proteins (Kajava et al., 2014; Daskalov, 2016). In addition, both signaling pathways (NLR-based amyloid signaling and necroptosis) led to PCD by plasma membrane damage through a conserved α-helical domain—named HeLo or HeLo-like in fungi and 4HB in metazoans (part of the necroptosis executioner protein MLKL) (Seuring et al., 2012; Hildebrand et al., 2014). Furthermore, the fungal HeLo and HeLo-like domains (and by extension the 4HB domain of MLKL) have homology with an RPW8-related CC_R_-type N-terminal domain of some plant NLRs, for which a membrane-targeting function in a context of innate immune response has also been reported (Collier et al., 2011; Wang et al., 2013; Daskalov et al., 2016; Zhong and Cheng, 2016). Thus, it seems likely that some PCD molecular pathways in all three major eukaryotic kingdoms have conserved mechanisms of cell death execution. Future studies are needed to understand the degree to which the molecular mechanisms have been conserved.

Taken together, these observations also point to a possible role in innate immunity for at least some fungal NLRs (Paoletti and Saupe, 2009). However, the large number of NLR-like proteins encoded in fungal genomes and their extreme architectural diversity suggests that this family of proteins may be involved in a variety of molecular responses, some of which may be important outside of the immune context and possibly not resulting in cell death.

## Concluding remarks

PCD in filamentous fungi has been identified as a crucial component of the invasion and colonization of host cells, for non-self-recognition mechanisms, during sexual and asexual differentiation and in aging. Additionally, PCD can be induced by treatment of fungi with a variety of chemicals and environmental onslaughts. These observations indicate that filamentous fungi have harnessed mechanisms of regulated death in response to a variety of factors, suggesting that multicellularity growth habits of these organisms have necessitated the evolution of PCD diversity. The multicellular nature of filamentous fungi vs. the unicellular lifestyle of yeast makes filamentous species particularly relevant models of PCD. This adds to the fact that a number of filamentous fungi are particularly easy to manipulate and maintain and because there is a wide assortment of available molecular, biochemical and genetic tools, they are very attractive options for the study of complex biological processes such as PCD. Some of the challenges that we predict will have to be overcome by researchers in the fungal PCD field include the discrimination of different cell death categories in fungi as well as their respective biochemical and morphological hallmarks. Nonetheless, the recent identification of molecular players in both recognition and execution of PCD has provided the groundwork for further investigations into both commonalities and differences in metazoan and fungal cell death.

## Author contributions

APG, JH, AD, AV, and NLG collectively wrote and edited the paper. JH constructed Figure 1, APG constructed Figure 2, and AD constructed Figure 3.

### Conflict of interest statement

The authors declare that the research was conducted in the absence of any commercial or financial relationships that could be construed as a potential conflict of interest.
